# Neural Correlates of Executive Control in the Avian Brain

**DOI:** 10.1371/journal.pbio.0030190

**Published:** 2005-05-10

**Authors:** Jonas Rose, Michael Colombo

**Affiliations:** **1**Department of Psychology, University of OtagoDunedinNew Zealand; University of EdinburghUnited Kingdom

## Abstract

Executive control, the ability to plan one's behaviour to achieve a goal, is a hallmark of frontal lobe function in humans and other primates. In the current study we report neural correlates of executive control in the avian nidopallium caudolaterale, a region analogous to the mammalian prefrontal cortex. Homing pigeons *(Columba livia)* performed a working memory task in which cues instructed them whether stimuli should be remembered or forgotten. When instructed to remember, many neurons showed sustained activation throughout the memory period. When instructed to forget, the sustained activation was abolished. Consistent with the neural data, the behavioural data showed that memory performance was high after instructions to remember, and dropped to chance after instructions to forget. Our findings indicate that neurons in the avian nidopallium caudolaterale participate in one of the core forms of executive control, the control of what should be remembered and what should be forgotten. This form of executive control is fundamental not only to working memory, but also to all cognition.

## Abstract 2

Recording from the forebrain of pigeons performing a working memory task, the authors find sustained neural activity during the memory period.

## Introduction

In 1861, Paul Broca [1] proclaimed that the “majesty of the human” could be attributed to its superior faculties, such as abstraction and judgement, and that these superior faculties lie within the province of the “anterior lobes of the brain.” Today the picture has changed little, and the frontal lobes, in particular the prefrontal cortex (PFC), are viewed as the repository of a host of higher-order faculties such as control of action, temporal organization of behaviour, sequencing, goal-directed behaviour, abstract and conceptual thinking, creativity, reasoning, and cognitive flexibility, to name a few [2–5]. Often these faculties are subsumed under two broad headings that define PFC function: *working memory* and *executive control.*

There is ample evidence that the PFC is important for both working memory, the ability to store information for short periods of time, and executive control, processes that operate on the contents of stored information. With respect to working memory, numerous studies in humans have shown that the PFC is activated during tasks that require retention of information [4,6,7]. Similarly, studies with monkeys have shown that certain neurons in the PFC display increased and sustained activation during the retention period of working memory tasks [8–11]. This sustained activation, also commonly referred to as delay activity because it occurs during the delay (memory) portion of a working memory task, is believed to represent a neural correlate of working memory [12–16].

In contrast to the evidence regarding the PFC and working memory, the evidence that the PFC is involved in executive control processes is considerably more varied in nature. In humans, tasks that require conflict resolution, that is, tasks that pit attending to task-relevant information and inhibiting task-irrelevant information, such as the Stroop Interference test or the Wisconsin Card Sorting Task, result in activation of the frontal cortex [17,18]. In monkeys, findings that PFC neurons are robust to distracting events [11], are modulated by attentional demands [19], encode abstract rules [20], and integrate information across different senses [21–23] have all been taken as evidence for its role in executive control processes.

Part of the reason for the varied evidence for executive control processes in both human and monkey studies is that the term *executive control* is itself somewhat poorly defined [24]. While the taxonomy of executive control still remains vague, some have argued that of all the processes that fall under the rubric of executive control, one of the fundamental processes is the ability to selectively remember relevant information and discard irrelevant information [18]. This seems reasonable, for although our memory capacity is impressive, it is not limitless [19,25,26], and we must possess and engage mechanisms that allow us to selectively filter information. In the current study, we provide evidence for neural correlates of this fundamental form of executive control in the avian brain.

We recorded from the nidopallium caudolaterale (NCL) of homing pigeons. On the basis of behavioural/lesion [27,28], neurochemical [29], and anatomical [30,31] studies, the NCL is considered, much like the PFC for mammals, to be the main integrative and executive area of the avian brain. The pigeons were trained on a directed forgetting [32,33] version of the delayed matching-to-sample task, a standard test of working memory used across a number of species, including birds, rats, monkeys, and humans. The birds were first shown a sample stimulus and then presented with either a remember cue or a forget cue (Figure 1A and 1B; see Materials and Methods). Following the remember or forget cue was a delay (memory) period. What followed the delay period was a function of whether the remember cue or the forget cue had been presented. If the remember cue was presented, then the delay was followed by a test period in which two stimuli were displayed, and a response to the stimulus that had appeared as the sample was rewarded. On the other hand, if the forget cue was presented, then following the delay there was no test period, and the trial terminated. Effectively, the remember cue instructed the bird that its memory for the sample would be tested after the delay and that it should therefore remember the sample stimulus, whereas the forget cue instructed the bird that there would be no test following the delay, and hence that it could forget the sample stimulus. Whether the cues were indeed instructing the birds to remember and forget the sample stimulus was tested with forget-probe trials (Figure 1C), which will be discussed shortly.

**Figure 1:**
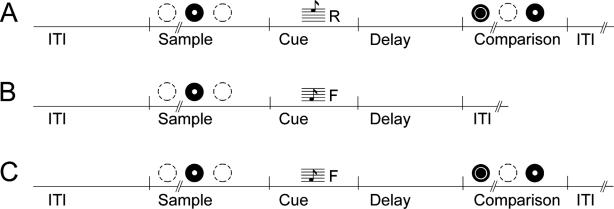
Behavioural Task Sequence of events on (A) remember trials, (B) forget trials, and (C) forget-probe trials. On remember and forget-probe trials the birds were presented with a test period, whereas on forget trials the test period was absent. The three horizontally arranged circles represent the projectors on which the stimuli, in this case a circle and dot, were displayed. During the cue and delay periods, the projectors were turned off. ITI, intertrial interval; R, remember cue, a high-frequency tone; F, forget cue, a low-frequency tone.

To the extent that sustained activation is a neural correlate of memory, and that the avian NCL is involved in executive control processes, we predicted that the activity of NCL neurons would be sensitive to cues to remember and forget. We found that neurons in the avian NCL showed sustained activation when the subject was instructed to remember, and the sustained activation was abolished when the subject was instructed to forget. Our findings indicate that neurons in the avian NCL participate in one of the fundamental forms of executive control, the control of what should be remembered and what should be forgotten.

## Results

### Histology

In two birds we recorded from both left and right NCL. For the three remaining birds, units were only recorded from either the left or right NCL. We recorded from a total of 124 NCL neurons. Figure 2 shows the electrode track reconstructions for the five subjects. With respect to the placements of the electrodes, the tracks were within the boundaries of NCL as defined by Kröner and Güntürkün [31]. All tracks were within 0.75 mm of the desired AP + 5.5 location (range AP + 5.25 to AP + 6.25), and all tracks were within 0.5 mm to the desired ML ± 7.5 location (range ML ± 7.0 to ML ± 8.0). We found no evidence that the characteristics of the neurons differed between the left and right hemispheres, along the dorsal and ventral extent of the NCL, or from one bird to the other. We have therefore collapsed the results across these variables.

**Figure 2:**
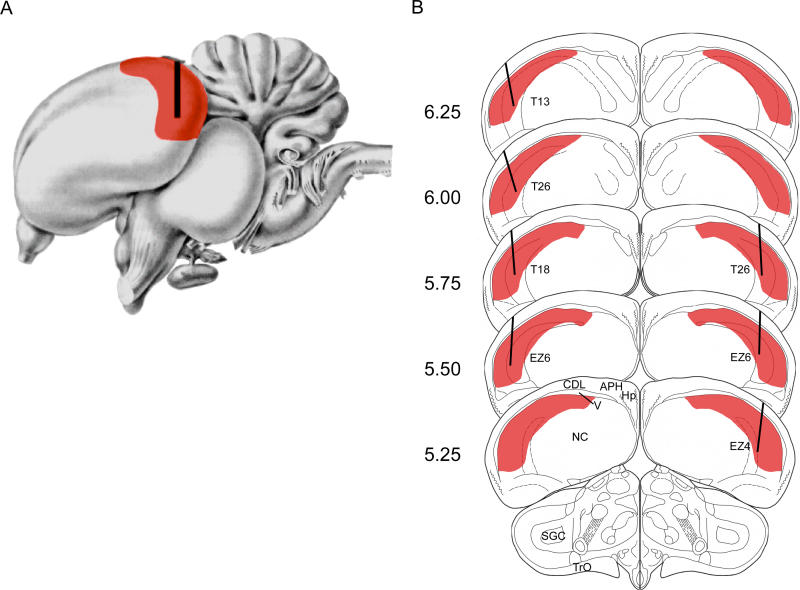
Histology (A) Lateral view of the pigeon brain. The NCL is shaded in red. The black line represents the intended electrode trajectory. (B) Histological reconstruction of the electrode tracks for the five pigeons. The NCL is shaded in red. The black lines represent the electrode track. All tracks were within the boundaries of the NCL. APH, area parahippocampalis; CDL, area corticoidea dorsolateralis; Hp, hippocampus; NC, nidopallium caudale; SGC, stratum griseum centrale; TrO: tractus opticus; V: ventricle.

### Incidence and Type of Delay Activity

A neuron was defined as a delay neuron if the level of activity during the delay period, when memory was required, was significantly different from the level of activity during the intertrial interval period, when memory was not required (paired *t*-test, see Materials and Methods, Data analysis). Of the 124 NCL cells, 83 (66.9%) were classified as delay neurons. For 30 of the 83 cells, delay activity occurred after only one of the two to-be-remembered (sample) stimuli used on the memory task; these cells therefore contributed one instance each of delay activity for subsequent analysis. The remaining 53 cells exhibited delay activity after both of the to-be-remembered stimuli; these cells therefore contributed two instances each of delay activity for subsequent analysis. In all, across the 83 delay cells there was a total of 136 instances of delay activity.

### Modulation of Delay Activity by Remember and Forget Cues

What effect did the remember and forget cues have on delay activity? In 120 of the 136 (88.2%) instances of delay activity, sustained activation was found only on remember trials, when memory was needed to solve the task. On forget trials, when no memory was required, the sustained activation was abolished. Examples of three neurons that exhibit this effect are shown in Figure 3. In each case, the remember cue was followed by a high level of activity that persisted beyond the cue period into and throughout the delay period. In contrast, the forget cue triggered an immediate decrease in activity during the cue period to intertrial interval (baseline) levels, and this decrease in activity persisted throughout the delay period. In short, following instructions to remember the sample stimulus, the cells exhibited sustained activation throughout the cue and delay periods, whereas following instructions to forget the sample stimulus, the sustained activation was abolished. Again, this effect was observed in the vast majority of instances of delay activity. Note that the pattern of data is not affected if we average the delay activity across the two sample stimuli and think of each cell as contributing only one instance of delay activity. In this case, of the 83 delay neurons, 63 (75.9%) showed the effect, that is, sustained activation on remember trials but not on forget trials.

**Figure 3:**
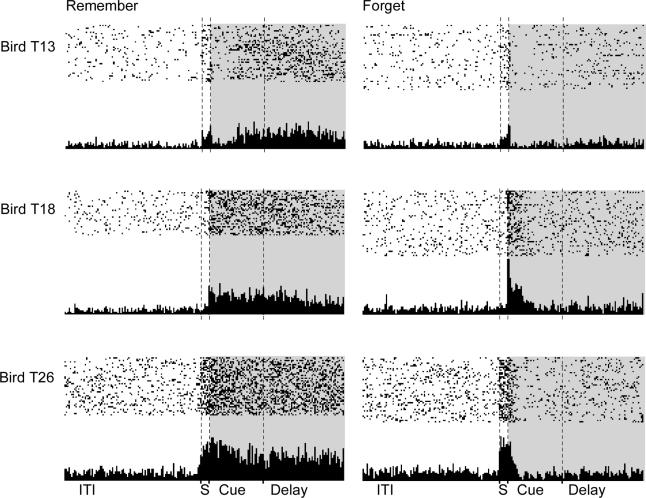
Modulation by Remember and Forget Cues Examples of remember and forget cues modulating neural activity in three delay neurons from three different birds. The cue and delay periods are shaded in grey. On remember trials, there is sustained activation in the cue and delay periods. On forget trials, the sustained activation is abolished. The binwidth is 50 ms. The vertical dashed lines separate the different periods of the task. ITI, intertrial interval; S, sample period.

### Population Response

Delay activity was further classified as excitatory or inhibitory, referring to an increase or decrease in activity relative to intertrial interval levels. Of the 83 delay neurons, 22 were inhibitory (10 contributing one instance of delay activity and 12 contributing two instances of delay activity) and 61 were excitatory (20 contributing one instance of delay activity and 41 contributing two instances of delay activity). In total, across the 136 instances of delay activity we encountered 102 instances of excitatory delay activity and 34 instances of inhibitory delay activity. The average response profile across all 102 instances of excitatory delay activity is shown in Figure 4. After a brief response to the remember cue, the population maintained high levels of activity for the rest of the cue and delay periods. In contrast, on forget trials, the response to the forget cue was followed by a drop in activity to baseline levels in the cue period, which persisted into the delay period. A repeated-measures ANOVA applied to all 102 instances of excitatory delay activity, with cue (remember vs. forget) and bins (100, 6, 40, and 60 bins of 50 ms in the intertrial interval, sample, cue, and delay periods, respectively) as factors, with repeated measures over bins, confirmed a significant difference in activity between remember and forget trials during the cue period, *F*(1, 202) = 24.3, *p* < 0.001, and the delay period, *F*(1, 202) = 16.4, *p* < 0.001, but not during the intertrial interval period, *F*(1, 202) = 0.07, *p* = 0.80, and sample period, *F*(1, 202) = 0.25, *p* = 0.62. A significant difference in activity in the delay period following remember and forget cues was also observed for the 34 instances of inhibitory delay activity, *F*(1, 66) = 5.4, *p* < 0.05. In these cases of inhibitory delay activity, the activity remained depressed after the remember cue, but was elevated to baseline levels after the forget cue.

**Figure 4:**
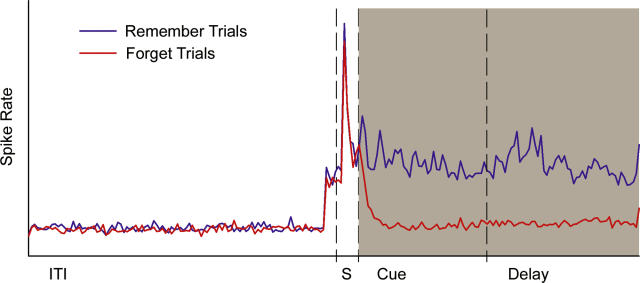
The Response Profile of Excitatory Delay Neurons The response profile of all 102 instances of excitatory delay activity on remember and forget trials. To account for differences in firing rates between the neurons, each neuron's firing rate was normalized against its baseline firing rate. The cue and the delay periods are shaded in grey. The vertical dashed lines separate the different periods of the task. ITI, intertrial interval; S, sample period.

### Behavioural Evidence of Forgetting

If sustained activation represents a neural correlate of active remembering, then the lack of such activity after the instruction to forget must be a neural correlate of active forgetting. The forget cue, however, acquires its function because it predicts the absence of the test period. If the birds are not tested after the forget cue, then how is it possible to know that the forget cue has directed them to forget the sample stimulus? We examined this issue by testing the birds occasionally with probe trials in which, against its prediction, the forget cue was followed by a test period. An example of such a forget-probe trial is shown in Figure 1C (see Materials and Methods). The birds were tested only rarely with these forget-probe trials. The reason for not testing more often with forget-probe trials is that, if we had done so, the ability of the forget cue to predict the absence of a test period would have been jeopardized.

Performance on sessions with forget-probe trials is shown in Figure 5A. On every one of the sessions, performance on the forget-probe trials was lower than performance on the remember trials. Figure 5B shows the performance on remember and forget-probe trials averaged across the four birds that were tested with probe trials. The average performance after the forget cue (43.8%) was significantly lower than the average performance after the remember cue (78.5%), *t*(3) = 3.31, *p* < 0.05. In addition, a one-sample *t*-test evaluated against 50% indicated that performance on forget-probe trials was not significantly different from chance, *t*(3) = 1.00, *p* = 0.39. The chance levels of performance on forget-probe trials indicates that the forget cue is indeed directing the subject to forget the sample stimulus, a finding in line with other studies using the directed forgetting paradigm [32,33].

**Figure 5:**
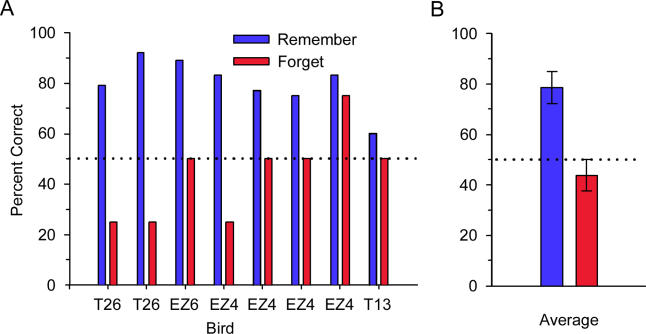
Performance on Probe Trial Sessions (A) The performance on each of the eight sessions with forget-probe trials. (B) The performance on the probe sessions (±1 SE) averaged across all four birds. The dotted line represents chance levels of performance.

## Discussion

### Summary of Findings

To summarize our findings, approximately 67% of the neurons we sampled in the NCL exhibited sustained activation during the delay period of the working memory task when the animal was required to engage memory processes. For the vast majority (88%) of the instances of delay activity, the sustained activation was modulated by instructions to either remember or forget. Instructions to remember resulted in sustained activation during the cue and delay portions of the memory task. In contrast, following instructions to forget, the sustained activation was rapidly abolished, and the neural activity returned to baseline levels. The high levels of performance following remember trials confirmed that the remember cue was directing the subject to remember the sample stimulus. In addition, the chance levels of performance on the forget-probe trials, in which the birds were cued to forget but then actually given a retention test, confirmed that the forget cue was directing the subject to forget the sample stimulus.

### Significance of Current Findings

The neurons in our study fired when the birds were told to remember, and they stopped firing when the birds were told to forget. We conclude that neurons in the avian NCL participate in one of the fundamental forms of executive control, the control of what should be remembered and what should be forgotten. Naturally, it is hard to know whether it is the NCL neurons that are performing the executive control function, or whether we are observing the effects on NCL neurons of executive functions that lie elsewhere in the brain. Given the evidence that we will review shortly that NCL may be an analogue of the mammalian PFC, and given the role of the PFC in executive control, we believe the former is the more likely case. This cause-and-effect issue aside, the need for such a filtering mechanism should be obvious. Although our memory capacity is impressive, it is not limitless [19,25,26]. We must have the ability, therefore, to filter information, allowing access to memory or retaining in memory that which is relevant, while restricting access to memory or discarding from memory that which is not.

Our data are the first example of neural correlates of executive control in a nonmammalian species. We would also argue that they are the most straightforward example of neural correlates of executive control reported in any species thus far. Using a delayed matching-to-sample task in which monkeys were cued to attend to certain visual information in an array of visually presented stimuli, Rainer et al. [19] found that the delay activity of PFC neurons was dominated by information from the attended rather than the unattended target. On the basis of these findings they concluded that “we hold in working memory that to which we attend” (p. 578). We take these findings one step further by showing that even after attending to a stimulus we can still make the executive decision to allow information to remain in working memory, or to discard that information from working memory. Our findings show that remembering and forgetting are both well controlled and active processes that can be engaged and disengaged at any time [16,34].

### Avian NCL and Mammalian PFC: Homologue or Analogue?

The NCL is a multimodal telencephalic region located in the posterior forebrain of birds. Divac and colleagues [28,29] were the first to suggest that the avian NCL might correspond to the mammalian PFC. Naturally, given almost 320 million years of independent evolution [35], there are some differences between the two structures. Most notably, the NCL is neither cortex nor in the frontal (i.e., anterior) part of the brain, but then topographical location is a poor criterion for comparing structures [36]. Although the NCL and PFC are not homologous structures [36], the evidence we review below does suggest that they are analogous structures.

Both NCL and PFC are ideally situated to integrate sensory information and translate that information into action. Anatomically, the NCL and PFC have similar patterns of afferent and efferent connections [31]. Both receive projections from modality-specific secondary visual, auditory, and somatosensory areas [31,37–39], and both project to motor and limbic areas of the brain [31,37]. In addition, both the NCL and PFC receive dense dopaminergic innervation from midbrain structures [29,40,41].

There are, of course, anatomical differences between the NCL and the PFC. One of the hallmark characteristics of PFC in primates is that it receives projections from the mediodorsal (MD) nucleus of the thalamus [42,43]. In birds, the main thalamic projection to the NCL is the nucleus dorsolateralis posterior thalami (DLP) [44]. The DLP, however, is not generally considered homologous to the MD, nor is it identical to the MD in all of its connections [27]. Nevertheless, there is evidence that the DLP may serve the same functions as the MD [27], and that therefore the DLP–NCL system may be analogous to the MD–PFC system.

There is also considerable behavioural/lesion data to support the contention that NCL and PFC perform similar functions. Damage to the NCL and PFC results in impairments on delayed alternation and pattern-reversal tasks while having little or no effect on simultaneous visual discriminations and basic sensory processes [2,27,28,45–47]. In addition, blockade of D1 receptors in NCL and PFC both cause impairments on tasks sensitive to PFC and NCL damage [48–50]. Finally, the response profiles of NCL neurons are similar to those found in PFC [51,52]. In summary, despite the anatomical and functional differences, which are not limited to comparisons between birds and mammals, but also include comparisons between one mammal and another [53], we believe there is considerable evidence to support the view that the avian NCL is analogous to the mammalian PFC.

### What Is Being Coded by Delay Activity?

Early studies of delay activity tended to be of the view that it represented a neural correlate of the to-be-remembered sample stimulus [12–14]. We have cast our findings along similar lines, and viewed the presence and absence of delay activity on remember and forget trials as neural correlates of the birds' remembering and forgetting the sample stimulus. The view that delay activity represents memory of the sample stimulus, however, has not gone unchallenged [54–57]. Recently, Lebedev et al. [58] have provided compelling evidence that delay activity, at least in PFC, represents more than just “maintenance memory.” By pitting attention and memory against each other, they reported that PFC delay activity more likely represents the location that the subject was attending to, rather than the location that the subject was remembering.

It is important to bear in mind that although we discuss our delay activity in terms of the subjects' remembering and forgetting the sample stimulus, there are other, equally likely, interpretations of the delay activity. For example, the remember and forget cues are also informing the subject about the prospect of obtaining a reward [59]: the remember cue tells the subject that there is the opportunity for a reward (assuming a correct response is made), and the forget cue tells the subject that no reward is forthcoming. The presence of sustained activation during remember trials, and the lack of such activity on forget trials, could therefore also reflect the neuron's coding for the possibility of a reward, and the absence of a reward, respectively. Similarly, along the lines suggested by Lebedev et al. [58], the remember and forget cues could also be informing the subject about where to direct attention in the chamber. Thus the presence and absence of sustained activation on remember and forget trials could therefore also represent a neural correlate of the subject attending to the spatial position where the choice stimuli will appear, and not attending to any particular spatial location, respectively.

The fact that sustained activation may serve as a code for the sample stimulus [52], aspects of the impending reward [60–62], or spatial attention [58] raises another important issue about PFC function. Many studies show that it is a combination of features that activate PFC neurons [61,62], a finding that may not be too surprising, given the known multimodal nature of the PFC [37,63], and a point that fits well with the established view that memory consists of a collection of attributes [64,65]. The point we are trying to emphasize is that even though delay activity correlates with aspects of the reward, attentional factors, and the memorandum, this does not weaken the notion that delay activity is a neural correlate of working memory, nor does it weaken the notion that the remember and forget cues are modulating the contents of working memory.

Against this backdrop of PFC neurons coding for attributes of working memory other than the sample stimulus, it might be interesting to speculate on our observation that the majority of NCL delay activity was nonselective in that it tended to occur after both (63.9%) rather than just one (36.1%) of the stimuli used on the memory task. A similar preponderance of nonselective delay activity has been observed in the hippocampus [15], which like the PFC, is also a zone of multimodal convergence in the brain [37]. In contrast, the majority of delay activity in the inferior temporal cortex, a higher-order area of the primate brain dedicated exclusively to visual processing, is selective, in that it tends to occur after only one rather than both of the stimuli used on the memory task [15]. It may be the case that delay activity in modality-specific visual areas may reflect more a code of the sample stimulus, whereas delay activity in multimodal areas like the PFC and NCL may reflect the other attributes of working memory, such as spatial attention and reward anticipation.

### The Value of Comparative Research

Is it necessary to invoke higher-order executive control mechanisms to explain the behaviour of pigeons in the current study? And in doing so, have we not dispensed with Occam's razor? We understand that it might be uncomfortable for some to accept that pigeons have executive control mechanisms. But if studies of animal cognition have shown us anything it is that behaviours once thought to be the exclusive domain of humans can be seen in many nonhuman (and nonmammalian) species [66]. The often heard retort to these examples of cognitive equivalencies is that just because you can mimic the behaviour of one species in another does not mean that the behaviour of both species is governed by the same mechanism. While true, the fact that you can get a bird to exhibit behaviour like that of a human could just as easily make us question how humans are solving the task, as make us question how birds are solving the task. The bottom line is that one would be hard pressed to argue against the view of mental continuity across species [66], and so if we accept executive processes in humans, it is very likely that executive processes exist in nonmammalian species as well.

What then are the implications of showing neural correlates of executive control in the avian brain? For one, it would appear that the ability to filter relevant and irrelevant information, what some have argued is a fundamental form of executive control [18], is clearly not an exclusive feature of the mammalian brain. However, we believe our data speak to another issue, one that emphasizes the importance of conducting comparative work. There is little doubt that it is commonplace to richly interpret human behaviour. But when you show that a pigeon can recognize itself in a mirror [67], or that a crow can manufacture a tool [68], it is not just an interesting demonstration, but it also constrains and refines our views regarding the neural mechanisms that underlie such behaviour. When you show that the avian hippocampus is in every respect an analogue of the mammalian hippocampus [69], yet at the same time structurally totally different [70], it is not just idle curiosity, but it also requires that we take this into consideration when we devise computational models of hippocampal function. And when you show that neurons in the avian brain engage in executive control processes, one must then wonder how much of what makes us such majestic creatures is present in an organism whose behaviour rarely inspires the attribution of high cognitive skills.

## Materials and Methods

### 

#### Subjects.

The subjects were 5 homing pigeons *(Columba livia)* weighing approximately 400–650 g. The animals were housed individually in wire-mesh cages inside a colony room, had free access to water and grit, and were maintained on a 12:12-h light:dark cycle with lights on at 0700. The pigeons were fed a mixture of wheat, peas, and corn in an amount adjusted to maintain them at 80% of their free-feeding body weight. The experiments were approved by the University of Otago Animal Ethics Committee and conducted in accordance with the University of Otago's Code of Ethical Conduct for the Manipulation of Animals.

#### Apparatus and stimuli.

All training and testing was conducted in standard sound attenuated operant chambers. Situated on the front panel of each chamber were three horizontally-arranged clear plastic circular response keys, each 2.5 cm in diameter. All three keys were mounted 22 cm above the floor and were 10 cm apart from centre to centre. Situated behind each key was a stimulus projector (IEE model 1071; Institution of Electrical Engineers, London, United Kingdom) used to deliver the visual stimuli. Food reward (wheat) was delivered via an illuminated magazine situated below the centre key. The stimuli consisted of standard geometric shapes (circle and dot) and two colours (red and white). The geometric shapes appeared as white forms against a black background. The circle was composed of a line that was 1.5 mm thick and was 17 mm in diameter, whereas the dot was solid and 7 mm in diameter. The colour stimuli consisted of illumination of the entire 25-mm diameter response key with the colour red or white. The presentation of the stimuli, reward, and punishment contingencies, and all data recording were controlled by Pentium II computers attached to the chamber.

#### Behavioural task.

The birds were trained on a directed forgetting version of the delayed matching-to-sample task. The sequence of events on a typical trial is shown in Figure 1. Each bird was trained with two stimuli, either a circle and a dot, or the colours red and white. All trials began with a 15-s intertrial interval, followed by the sample period, during which one of two stimuli was displayed on the centre of three keys. Following three pecks to the sample stimulus, the sample was turned off. This was followed by a cue period in which either the remember or forget cue was presented for 2 s. A delay period of 3 s followed the cue period. If the remember cue had been presented (Figure 1A), the delay was followed by a test period in which both stimuli were displayed. A single response to the stimulus that had been presented in the sample period was rewarded with wheat, whereas a single response to the other stimulus was punished with a 30-s time-out. If the forget cue had been presented (Figure 1B), the trial ended after the delay period without a test period.

A session consisted of 96 trials, 48 remember trials and 48 forget trials, randomly mixed. With two stimuli (A and B), there are four possible trial types: sample A can be followed by A on the left and B on the right, or B on the left and A on the right, likewise for sample B. Which of the two stimuli (e.g., A or B) was presented during the sample period, the position of the two stimuli on the side keys during the test period (e.g., A B or B A), as well as the sequence of remember and forget cues, was balanced within a session. For different birds, the remember and forget cues were either a high-frequency tone (5,000 Hz) and a low-frequency tone (500 Hz), or a four-lobed visual pattern (two figure eights superimposed on each other) and a 16-lobed visual pattern (resembling a sun). The four-lobed and 16-lobed patterns appeared as white shapes against a black background and were each 17 mm in diameter.

Approximately after every 15th recording session the birds were tested with forget-probe trials (Figure 1C). These forget-probe trials were administered to four of the five birds; bird T18 did not receive a probe trial session because we lost the ability to record from this bird after the 11th recording session. These probe trials allowed us to examine the effectiveness of the forget cue as an instruction to forget the sample stimulus. Recall that on standard forget trials, following the delay period there was no test period, so it is impossible to know whether the subject had indeed forgotten the sample stimulus. On a forget-probe trial, against its usual prediction, the forget cue was followed by a test period. A probe trial session contained four forget-probe trials in addition to the 48 remember and 48 forget trials. All aspects of the stimulus and cue presentations were balanced within a session.

#### Training protocol.

Although all of the birds had been trained on a simultaneous matching-to-sample task, none had received any delayed matching-to-sample experience at the start of this experiment. Delayed matching-to-sample training was divided into two phases. In phase 1, the birds were first trained with a 0-s delay followed by training with a 0.5-s delay. The birds were required to achieve a performance level of two consecutive sessions at or better than 90% correct at each delay. To complete phase 1 the birds required an average of 43 sessions (range 24–63 sessions) and accumulated an average of 1,987 trials (range 1,297–3,264 trials).

In phase 2 the birds were introduced to the directed forgetting procedure, which used the same delayed matching-to-sample task but with remember and forget cues. The birds were gradually trained with increasing delays and increasing session lengths until they were able to perform the task with a 5-s delay (2-s cue period and 3-s delay period) and 96 trials per session. To complete phase 2 the birds required an average of 214 sessions (range 148–236 sessions) and accumulated an average of 14,545 trials (range 11,088–16,268 trials) on the task. Note that half of the trials accumulated during phase 2 were forget trials.

#### Surgery.

Upon completion of behavioural training, the birds were prepared for alert recording by implanting a miniature movable microdrive [71]. Surgery was conducted under ketamine (225 mg/kg) and xylazine (5 mg/kg) anaesthesia. The head was immobilized using a Revzin stereotaxic adapter [72]. A topical anaesthetic (10% Xylocaine) was applied to the scalp, which was then cut and retracted to expose the skull. A small hole above the NCL was drilled through the skull at AP + 5.5 and ML ± 7.5 [72], and the microdrive then lowered so that the tips of the electrodes were positioned just above the NCL. Stainless steel skull screws, one serving as a ground screw, were placed into the skull, and the entire microdrive was attached to the skull using dental acrylic. The incision was then sutured, Xylocaine applied to the wound margin, and the animal allowed to recover in a heated, padded cage until alert and mobile, at which point it was returned to its home cage. All animals were allowed to recover for 7–10 d prior to the start of recording.

#### Neuronal recording.

The microdrive housed eight 25-μm Formvar-coated nichrome wires that were used to measure the extracellular activity of single neurons. All signals were first impedance matched through a FET headstage and then amplified and filtered to remove 50 Hz noise using Grass P511K preamplifiers (Grass Instruments, Quincy, Massachusetts, United States). A separate electrode with minimal activity served as the indifferent electrode. The signals were monitored with an oscilloscope and speaker. Behavioural time-tagging of all events and analysis of the spike data was accomplished using a CED 1401 plus system (Cambridge Electronic Design Limited, Cambridge, United Kingdom) and CED Spike 2 software. The only criterion for the selection of a neuron was that it was well isolated with a signal-to-noise ratio of at least 2:1. After isolating a neuron, the delayed matching-to-sample task was started. A typical session lasted approximately 45 m to 1 h. The pigeons were tested once a day. At the end of the recording session, the electrodes were advanced at least 40 μm and the animal returned to its home cage.

#### Histology and electrode track reconstruction.

Upon completion of the experiment, the final electrode position was marked by passing a current through each electrode, thus creating a small electrolytic lesion. The pigeons were then deeply anaesthetised with halothane and perfused through the heart with physiological saline followed by 10% formalin. The brains were blocked, removed, placed in 10% formalin for 5 d, placed in 30% sucrose and 10% formalin, and allowed to sink twice. The brains were then frozen and sectioned at 50 μm, with every section mounted and stained with cresyl violet. The positions of the recorded neurons were calculated from the electrode track reconstructions, position of the electrolytic lesion, and depth records.

#### Data analysis

Only correct trial data were analyzed. For initial assessment of delay activity, for each neuron the average activity in the middle 5 s of the intertrial interval was compared to the average activity in the delay period after each of the two sample stimuli using paired *t*-tests with a modified [73] Bonferroni correction (*p* < 0.025). The population data were subjected to a repeated-measures ANOVA with Greenhouse–Geisser correction.

## Abbreviations

DLP: dorsolateralis posterior thalami
MD: mediodorsal
NCL: nidopallium caudolaterale
PFC: prefrontal cortex
